# One Hundred Consecutive Cases of Skin Disease

**Published:** 1891-09

**Authors:** M. B. Hutchins

**Affiliations:** Lecturer on Diseases of the Skin, Atlanta Medical College, Atlanta, Ga.


### ONE HUNDRED CONSECUTIVE CASES OF SKIN DISEASE.

### VIII.

BY M. B. HUTCHINS, M. D.,
Lecturer on Diseases of the Skin, Atlanta Medical College, Atlanta, Ga.

### EPITHELIOMA.

In the hundred cases of skin disease there were two cases of epithelioma.

First, on arm of gentleman aged 67. Growth was situated on flexor surface left forearm, and began several months previously as a "pimple," irritation of shirt wristband gradually producing condition to be described. There was a raised, hard infiltration, confined to the skin, circumscribed, size of the finger nail and of reddish color. In the centre was a rather "sharp cut," deepish "ulcer," size of a pea, secreting a little thin, serous fluid, and showing a few dilated vessels in base. A small, hard papule just inside upper border. A few months later ulceration had ceased, but the growth continued to enlarge, retaining the characteristic hard, waxy border. Finally the epithelioma was excised by the patient's family physician, and it is scarcely possible that there will be a recurrence.

Second case was that of a gentleman aged 60. Disease situated on right side of neck along line of contact of collar. Began nine years preceding as a "little chafed place." When first seen by me the growth was about the size and shape of a finger, transverse to the neck, and formed of hard infiltration of the skin, of waxy appearance. At points it was papillary (the papular growths possessing the characteristic hardness); in other parts there were to be seen, at intervals, ulcerations elliptical in shape, base covered with "blood" crusts, edges hard; seat of destruction bled easily. Sensations were of tingling and itching. No involvement of lymphatic glands. On right cheek was a round, infiltrated, scaly lesion, and on left side of neck a somewhat similar formation, neither larger than a finger nail. Excision of growth on neck was advised, but the patient, so far as I know, has never had the operation done, being content because he found that the growth was looking better since it had been protected from rubbing of collar. The general health of both these patients was robust.

### VARICOSE ULCER.

There was only one case of this "trouble." Patient single woman, employed as house-keeper, age 35. On left leg, just above ankle, inner side, two superficial, irregular, thinly-crusted ulcerative lesions, surrounding skin pigmentated. Patient made the remarkable statement that this condition had been present "off and on" since she was thirteen years old! All the superficial veins of this leg and the foot were enormously dilated, patulous, and some felt hard and resistant. There were extravasations in skin of instep.

Ordered to keep leg well bandaged from toes to knee, discard the garter, keep foot elevated when possible, and keep the ulcers wet with

R. Ichthyol., ʒi.

Aquæ, ℥iv.

M.

There was steady improvement. Later, when there was only a little "tenderness," and slight crusting or scaling, the treatment was varied by the use of

R. Ichthyol., gr. xv.
Ungt. zn. ox., ℥i.
M.

Under this treatment the ulcers were thoroughly healed, despite the terrible condition of the veins and the patient's unalterable conviction that the ulcers were the result of "blood disease."

While in the Skin and Cancer Hospital in New York I saw this plan invariably succeed, and have further proven its efficacy in cases treated in the "*second hundred.*"

TINEA VERSICOLOR.

The only case of this disease was that of a medical student aged 23. Disease began ten years before, and in the past two years had rapidly involved the whole upper part of chest. There was evidence of the disease having formerly been in discrete patches, but now these patches had formed a confluent covering of the surface. Around borders were still to be seen separate small, typical maculæ, probably marking further spread of disease. Affected skin normal in thickness, patches yellowish in color, becoming red when rubbed. Thin branny scales easily rubbed off. Itching when patient became heated. A recent attack of measles had no apparent effect on the disease. Has never been treated. Under the microscope the scales were seen to be almost entirely composed of the fungus known as "*microsporon furfur*"

Patient was directed to "*scrub*" affected surface well with plain soap and water and then apply

R. Hydrarg. bichlor., gr. xx.
Glycerin, ʒii.
Sp. vin. rect.,
Aquæ, aa ℥ii.

M.

I did not see the case again, as patient left the city in a few days.

### SCLERODERMA.

The one case of this disease was published in the JOURNAL last year.

### SYCOSIS.

Of "true," or "non-parasitic," sycosis there was one case—young man of 28. Duration, "better or worse," five years. On left cheek in beard, in hair back of neck and on upper lip inflammatory papules and pustules each pierced by a hair and surrounded, or separated from each other, by healthy skin. (The above description gives, "in a nutshell," the diagnostic characters of the disease.) Later there were a few "abortive" lesions outside the hairy area.

An ointment with three per cent. ichthyol had no effect. Epilation of hairs involved was practiced from the beginning of treatment. Disease gradually declined under bathing face in water, to which patient was directed to add a little bichloride of mercury (he had access to, and knowledge of, drugs), and the use of

R. Hydrag. bichlor., gr. i.

Sp. vin. rect., ʒii.

Ungt. zn. ox., ℥i.

M.

Treatment was concluded with

R. Acid. salicyl., gr. x.
Ungt. diachyli, ℥i.

M.

Discharged, well, in a month. Some months later there was a relapse, which I did not treat.

NÆVUS VASCULOSUS.

One case, little girl of 6 years. Beneath right orbit a horizontal, small straw sized dilated capillary, half inch long and radiating from this short capillary branches, all the vessels arterial red. (This corresponds to the old "nævus araneus," or "spider nævus"). Her father said the nævus had formed within the year. Destruction by electrolysis was advised, but the patient did not return.

CARCINOMA CUTICULARE.

This case was published in *New York Journal of Cutaneous and Genito-Urinary Diseases* in May of this year, and reprinted in this JOURNAL for July.

ERYTHEMA MULTIFORME.

Of this disease there was one case—that of a physician age 34. Duration four months. On forearms, wrists, thighs, at occipito-cervical junction, glans penis, abdomen and lower legs were grouped or solitary papular lesions, yellowish in color, average size small finger nail. Some were in rings. Some of the rings had coalesced to form gyrate patches. Older lesions slightly scaly; the yellowish or fawn color marked in all. Larger were depressed in the centre, the depressed part paler than the border. Sensations of slight itching. At beginning the patient had poor appetite, with "slight chilliness and fever at night."

Ordered to continue internally his own prescription of iron, quinine and strychnine and to paint the lesions with (the now familiar)

R. Picis. liq., ʒi.

Ether sulph.,

Sp. vin. rect., aa ℥ss.

M.

When, at end of three weeks, patient was seen, the eruption had disappeared. Had relapse later, which got well under same treatment in two or three weeks. No recurrence since.

### MOLLUSCUM EPITHELIALE.

The records of this case are not complete, as the patient returned to have treatment finished up after an absence of about eight months. As the disease is one of considerable interest, this case will probably be reported in detail when the treatment is finished.

### "CINDERS IN SKIN."

This descriptive title was given to the case of a railroad man who had been thrown upon his face in a heap of cinders, in an accident. The skin around and above the right orbit was injured by the cinders, and particles were embedded which remained after healing of wounds, producing blue-black discolorations like tattooing. From time to time inflammatory action would take place, causing extrusion of some of the particles, Nature's effort at cure. A few of the colored lines were treated by an effort to imitate Nature. A silk thread was passed through the length of the line, tied and left to produce all the suppuration possible. The effect was much as desired, but the patient became annoyed with the threads and discontinued their use.

It was found impossible to pick out the deeply embedded particles without leaving an ugly scar. Two or three of the discolored lines over the orbit were excised, and also a large and very disfiguring "spot" in the soft skin of lower lid just at margin of orbit. Here a triangular piece of skin, one-third of an inch from point to point, was removed and the edges of the wound so brought together as to entirely prevent ectropion.

The excised skin was hardened in alcohol and sections for microscopic examination were made. The minute cinder particles were found thoroughly incorporated in the connective tissue of the corium, as if they were a part of its structure, showing how futile would have been further attempts at "picking out."

### VERRUCA–WARTS.

The case coming under this head was that of a little negro having a palm-sized patch composed of closely set warty growths just below the left knee. I think this case of sufficient interest to be published later, together with some cases in which the warty growths were also prominent, but will say a complete cure was made in a month with

R. Acid. salicyl.
Sp. vin. rect.

M. Ft. *Solutionem saturatum.*
Sig.—" Paint " on and let dry. Then rub in.

(*Xeroderma pigmentosum, etc., next article.*)

1½ *Edgewood Ave.*

---

SPRAINS AND RHETORIC.—The man that will give to the world an unfailing remedy for sprains shall have his name writ high upon the wall of the temple of fame, and his praises shall be sung through long ages by the bards of a grateful humanity.—*Med. Record.*

---

Medicine is the noblest of professions ; the meanest of trades. Unless you can live lives of purity, of virtue, of honor, and of honesty, seek a livelihood elsewhere, and insult not the gods by striving through base methods and ignoble ambitions to resemble them.—*T. Gaillard Thomas.*

---

From a private communication we learn that for some reason or other, climatic, meteorological, or sociological, the section of country around Rock Rum, Alabama, is not very conducive to large families. The cause of midwifery is languishing from disuse. The people do not obey the divine injunction, to be fruitful and multiply. At any rate, the children seem to be very unevenly distributed. One gentleman is the proud father of twenty-eight children, and our informant tells us that he looked as if he might continue to multiply and replenish the earth for many years yet.

