# Erratum

**Published:** 1891-10

**Authors:** 


*ERRATUM.—In article VIII. "Carcinoma *Cuticulare*" should have been printed, Carcinoma *Lenticulare*.

